# Fetuin-A and Fetuin-B in Non-Alcoholic Fatty Liver Disease: A Meta-Analysis and Meta-Regression

**DOI:** 10.3390/ijerph17082735

**Published:** 2020-04-15

**Authors:** Xiongfeng Pan, Atipatsa C. Kaminga, Jihua Chen, Miyang Luo, Jiayou Luo

**Affiliations:** 1 Xiangya School of Public Health, Central South University, Changsha 410008, China; pxfcsu@163.com (X.P.); atipatsachiwanda@yahoo.co.uk (A.C.K.); chenjh@csu.edu.cn (J.C.); 2 Department of Mathematics and Statistics, Mzuzu University, Mzuzu 999120, Malawi; 3 Saw Swee Hock School of Public Health, National University of Singapore, Singapore 999002, Singapore

**Keywords:** fetuin-A, fetuin-B, non-alcoholic fatty liver disease, meta-analysis, meta-regression

## Abstract

The magnitude of the effect of fetuin-A and fetuin-B on non-alcoholic fatty liver disease (NAFLD) remains undefined. Therefore, the aim of this study was to synthesize previous findings to obtain a reliable estimation of this relationship. This study was registered in PROSPERO with the number CRD42019126314. Studies published not later than March 2019, examining the relationship between fetuin-A, fetuin-B, and NAFLD, were identified by a systematic search in the electronic databases of the Web of Science, PubMed, Embase, and Cochrane Library. Pooled estimates of standardized mean difference (SMD), calculated using the random-effects model in a meta-analysis, were applied to estimate the strength of the association between fetuin-A, fetuin-B, and NAFLD. Thirty publications were identified and analyzed based on specified inclusion criteria. Collectively, they consisted of 3800 NAFLD participants and 3614 controls. Compared with the controls, significant higher values of the fetuin-A (SMD = 0.83, 95% CI: 0.59 to 1.07, Z = 6.82, *p* < 0.001) and fetuin-B (SMD = 0.18, 95% CI: 0.02 to 0.33, Z = 2.27, *p* = 0.023) were observed in NAFLD patients. Meanwhile, in the subgroup analysis, the effect value of fetuin-A in the NASH group was significantly higher than that in the NAFL group (*p* = 0.036). The findings of this study suggest that elevated fetuin-A and fetuin-B may independently indicate the occurrence of NAFLD. Nevertheless, further research is needed to confirm these results.

## 1. Background

Non-alcoholic fatty liver disease (NAFLD) is now highly prevalent and often related to complications such as liver failure, cirrhosis, and liver cancer. Thus, it has become a major health concern and new worldwide epidemic. Basically, NAFLD is a chronic hepatic steatosis from non-alcoholic fatty liver (NAFL) to non-alcoholic steatohepatitis (NASH) [1]. However, its exact pathogenesis is still obscure. Notwithstanding, there has been increasing evidence to indicate that hepatokines such as fetuin-A and fetuin-B may influence the prognoses of NAFLD and associated complications [2]. Besides, epidemiological investigations also showed that fetuin-A and fetuin-B not only played a role in the development of obesity, hyperglycemia, and hypertriglyceridemia, but also led to chronic diseases, such as metabolic syndrome (MetS), type 2 diabetes mellitus (T2DM), and cardiovascular disease (CVD) [3,4]. In addition, current evidence in genetics suggests that, compared with the healthy controls, patients with liver steatosis have higher expression of fetuin-A mRNA in hepatocytes and serum fetuin-A concentrations [5]. The different amounts of this protein in the two groups could be due to the fact that fetuin-A (a2HS-glycoprotein), which is a glycoprotein produced by the adipose tissue and liver, has a dual function, that is, it restrains the insulin receptor and brings about insulin resistance in the pancreas, skeletal muscle, and hepatocytes [6]. It also has an inverse relationship with tumor necrosis factor (TNF-α), interleukin-6 (IL-6), and C-reactive protein (CRP) level, which is the critical factor in systemic inflammation in NAFLD [7]. However, unlike fetuin-A, fetuin-B is secreted by hepatocytes of a high-fat diet and does not induce pro-inflammatory signals and macrophage activation. Rather, it increases in steatosis and induces insulin resistance. In addition, experimental studies have shown that serum levels of fetuin-B are not associated with adiponectin levels. Although some previous studies indicated that both fetuin-A and fetuin-B belonged to the proteins attributed to the cystatin superfamily with shares of 22% sequence similarity, other studies revealed the differential effects of fetuin-A and fetuin-B as regards to the pathophysiology of fatty liver [8]. For example, the concentrations of fetuin-A and fetuin-B have been shown to be positively correlated and this relationship played a role in NAFLD, even after controlling for gender and age [8].

To sum up, there is clear evidence that fetuin-A and fetuin-B are complex metabolic phase reactants that have controversial roles in NAFLD. Therefore, the aim of this study was to synthesize previous findings to obtain a reliable estimation of the relationship between fetuin-A, fetuin-B, and NAFLD.

## 2. Methods

### 2.1. Search Strategy

This study was carried out using the guidelines provided in the Cochrane handbook (version 5.1.0) for systematic reviews and meta-analysis, whereas the results were reported according to the Preferred Reporting Items for Systematic Reviews and Meta-Analyses (PRISMA) [9]. In addition, this study was registered with the International Prospective Register of Systematic Reviews PROSPERO (CRD42019126314) by XP. 

Studies published between January 1959 and March 2019, examining the relationship between fetuin-A, fetuin-B, and NAFLD, were identified by a systematic search in the electronic databases of the Web of Science, PubMed, Embase, and Cochrane Library. Detailed search strategies were designed with help from experienced librarians and are shown in the appendix of the Supporting Information. Details of the search strategy are presented in Supplementary File. For example, in the PubMed database, the literature search was carried out using the following search strategy:

((alpha 2 HS Glycoprotein[Title/Abstract] OR alpha(2) HS Glycoprotein[Title/Abstract] OR alpha-2HS Glycoprotein[Title/Abstract] OR alpha 2HS Glycoprotein[Title/Abstract] OR alpha2HS Glycoprotein[Title/Abstract] OR Fetuin-A[Title/Abstract] OR Fetuin-A[Title/Abstract] OR AHSG Protein[Title/Abstract] OR alpha2 Heremans-Schmid Glycoprotein[Title/Abstract] OR alpha-2-HS-Glycoprotein[Title/Abstract] OR fetuins[Title/Abstract] OR Fetuin[Title/Abstract] OR Fetuin-B[Title/Abstract] OR Fetuin-B[Title/Abstract])) AND (Non-alcoholic Fatty Liver Disease OR NAFLD OR Nonalcoholic Fatty Liver Disease OR Fatty Liver, Non-alcoholic OR Fatty Livers, Non-alcoholic OR Liver, Nonalcoholic Fatty OR Livers, Nonalcoholic Fatty OR Nonalcoholic Fatty Liver OR Nonalcoholic Fatty Livers OR Nonalcoholic Steatohepatitis OR Nonalcoholic Steatohepatitides OR Steatohepatitides, Nonalcoholic OR Steatohepatitis, Nonalcoholic OR Non-alcoholic Fatty Liver Disease OR non-alcoholic steatohepatitis OR NASH OR Fatty Liver OR non-alcoholic steatohepatitis OR non-alcoholic fatty liver disease).

### 2.2. Eligibility Criteria

Titles, abstracts, and full texts of the studies yielded after searching in the electronic databases were screened to meet the following inclusion criteria: (1) Studies were case-control studies; (2) studies reported a criteria used to diagnose NAFLD; (3) studies provided mean and standard deviation (SD) of fetuin-A and fetuin-B, or these could be obtained from respective authors on request; (4) studies were peer-reviewed before publication; and (5) studies were published in English. On the contrary, the exclusion criteria were: (1) Studies were case reports or reviews; (2) studies reported NAFLD in combination with other diseases; (3) studies had fetuin-A and fetuin-B pharmacologically challenged before their measurements; (4) studies did not examine humans; and (5) studies were grey literature (unpublished literature). Two researchers [KA and AK] independently identified the eligible studies, and their discrepancies were resolved by involving the third researcher [JL]. 

### 2.3. Data Extraction

Data related to the following variables were extracted: (1) The first author’s name and year of publication; (2) subject’s characteristics such as Body Mass Index (BMI), mean age (mean, SD), and gender; (3) region of study; (4) participant’s laboratory characteristics such as aspartate aminotransferase (AST), alanine aminotransferase (ALT), glutamyl transpeptidase (GGT), low-density lipoprotein (LDL), high-density lipoprotein (HDL), triglycerides (TG), and insulin resistance index (HOMA-IR); (5) sample characteristics such as the material of sample, and mean and SD of fetuin-A and fetuin-B; and (6) fetuin-A and fetuin-B sample test methods, and storage temperatures. Two researchers [KA and AK] conducted data extraction independently using EpiData 3.0 and Microsoft Excel 2007. Any discrepancies between them were resolved by consensus.

### 2.4. Quality Evaluation

The non-randomized Newcastle-Ottawa Scale (NOS) was used to assess the quality of this study [10]. It is a 9-star rating system designed for non-randomized studies, which includes 3 domains and 8 items. The 3 domains consisted of the following broad perspectives: (1) Selection; (2) comparability; and (3) outcome. According to the NOS, study quality was classified as low, moderate, and high according to the scores, 0–3, 4–6, and 7–9, respectively.

### 2.5. Statistical Analysis

The random effects model was used to pool the estimates under discussion because of the varying populations and criteria used to define outcomes across the eligible studies. This model, therefore, was used to merge standardized mean differences (SMDs) and corresponding 95% confidence intervals (CIs), to compare the concentrations of fetuin-A and fetuin-B between NAFLD patients and the controls, which was calculated as Cohen’s d [11]. The pooled effect value, SMD, was considered to be high if greater than 0.8, moderate if between 0.5 and 0.8, or low if lower than 0.5. In addition, heterogeneity between-study was assessed using the Cochrane Q statistic, and the level of heterogeneity was measured by the I^2^ statistic [12]. Similarly, the heterogeneity was considered low if the I^2^ statistic was lower than 25%, moderate if it was 25%–75%, and high when it was greater than 75%. Additionally, the overall results were divided into the subgroups, including NASH patients and the control group, and NAFL patients and the control group. In addition, subgroup analysis was conducted to explore whether samples, storage methods, or ethnicity had any influence on the results. However, the subgroup analysis of fetuin-B was not carried out due to inadequate information related to fetuin-B. Furthermore, meta-regression analyses were conducted to explore the source of heterogeneity when this was significantly high. Subjects’ characteristics such as BMI and mean age (mean, SD); and participant’s laboratory characteristics such as AST, ALT, GGT, LDL, HDL, and TG were taken into account when exploring the source of heterogeneity. Additionally, a sensitivity analysis was performed by repeating the meta-analysis when each was omitted in turn. Publication bias was assessed by examining the symmetry of funnel plots when the number of studies reporting the primary outcomes was at least 10, and the significance of the symmetry was tested using the Egger’s linear regression test [13]. All analyses were done with the ‘meta’ and ‘metafor’ packages in R software (version R 3.4.3, MathSoft, Englewood, NJ, USA). In addition, all the statistical tests were two-sided, and *p*-values less than 0.05 indicated significant results for all statistical tests.

## 3. Results

### 3.1. Literature Search

**Figure 1:**
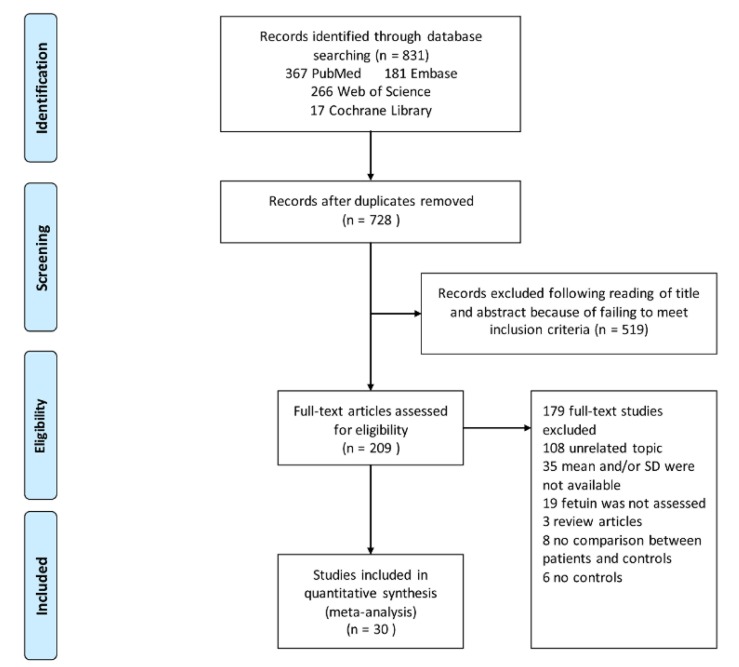
**Flowchart of study selection.** Showing the process by which retrieved studies from the databases were assessed and selected or excluded. Preferred reporting items for systematic reviews and meta-analyses (PRISMA) diagram for study search.

Figure 1 shows an overview of the search results and screening process. A total of 831 articles were found in the four electronic databases as follows: 181 from Embase, 17 from the Cochrane Library, 266 from Web of Science, and 367 from PubMed. After excluding the duplicates, 728 articles were retained. The titles and abstracts of the 728 articles were reviewed, and this resulted in 519 articles excluded because they did not meet the inclusion criteria. Then, a full-text review of 209 articles further excluded 179 studies, of which 108 were unrelated to the topic of this study, 19 had no data on fetuin-A and fetuin-B, 35 did not report means and SDs of fetuin-A and fetuin-B concentrations, 8 did not compare fetuin-A and fetuin-B concentrations between NAFLD patients and a control group, 3 were reviews, and 6 did not report the results of a control group. Finally, a total of 30 articles met the inclusion criteria and were included in the final meta-analysis.

### 3.2. Characteristics of Eligible Studies

**Figure 2:**
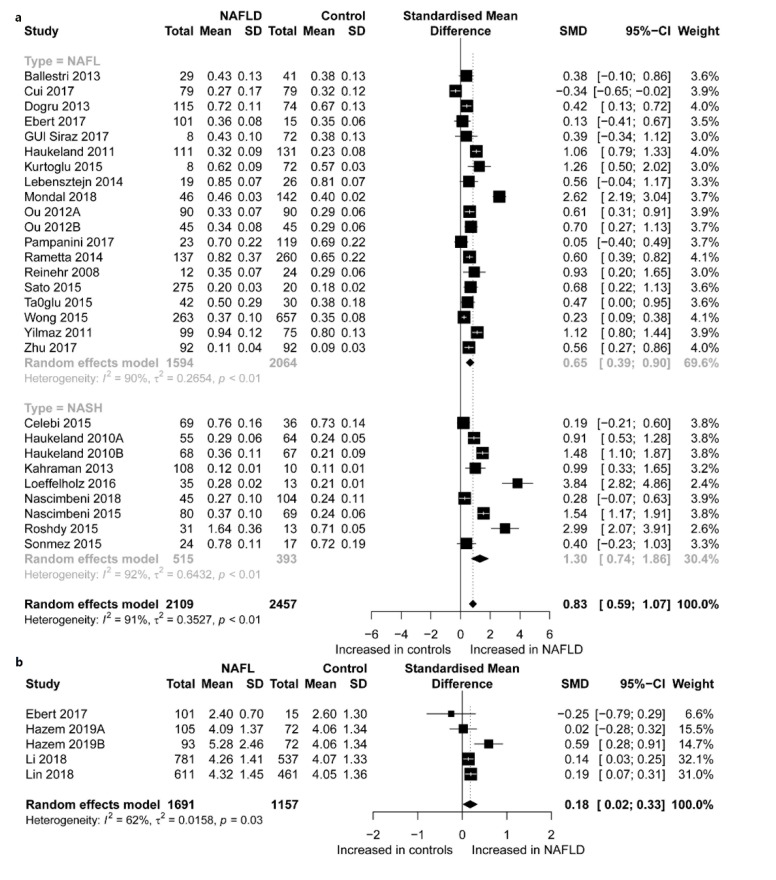
Forest plots of the standardized mean differences (SMDs) of fetuin-A concentrations (**a**), and fetuin-B concentrations (**b**) between the NAFLD participants and controls. The SMDs represent the effect sizes of fetuin-A, and fetuin-B concentrations on NAFLD. Each data marker represents a study, and the size of the data marker is proportional to the total number of individuals in that study. The summary effect sizes of fetuin-A, and fetuin-B concentrations are denoted by a diamond sign. The abbreviations in the forest plots are translated as follows: NAFLD, non-alcoholic fatty liver disease; NASH, non-alcoholic steatohepatitis; NAFL, non-alcoholic fatty liver; SMD, standardized mean difference.

Characteristics of the 30 eligible studies are presented in Table 1, which shows the patients’ characteristics such as age, gender, BMI, and region; sample characteristics such as the source of the sample, and mean and SD of fetuin-A and fetuin-B concentrations. Characteristics of clinical parameters of the studies included for the meta-analysis of fetuin-A, fetuin-B, and NAFLD are presented in Table 2. Participant’s laboratory characteristics such as AST, ALT, GGT, LDL, HDL, HOMA-IR, and TG; fetuin-A and fetuin-B sample assay methods; and storage temperatures. A total of 28 analyses of fetuin-A (Figure 2a) and 5 analyses of fetuin-B (Figure 2b) were identified from the 30 eligible studies. Altogether, these studies compared fetuin-A concentrations between 2109 NAFLD patients and 2457 controls; and fetuin-B concentrations between 1691 NAFLD patients and 1157 controls. The NOS scores of these studies varied between 5 and 8, with 21 studies graded as high quality and 9 as moderate quality.

### 3.3. Overall Comparison

Figure 2 presents forest plots of the standardized mean differences (SMDs) of fetuin-A and fetuin-B concentrations between the NAFLD participants and controls. Overall, taking the studies which compared fetuin-A concentrations between patients with NAFLD and the controls, it was found that fetuin-A concentrations were significantly higher in NAFLD patients than in the controls, with high effect (SMD = 0.83, 95% CI: 0.59 to 1.07, *p* < 0.001), and heterogeneity was considerable (I^2^ = 91.1%). In addition, subgroup analysis found that fetuin-A concentrations were significantly higher in the NAFL patients than in the controls, with high effect (SMD = 0.65, 95% CI: 0.39 to 0.90, *p* < 0.001); and in the NASH patients than in the controls, with high effect (SMD = 1.30, 95% CI: 0.74 to 1.86, *p* < 0.001), but both with high heterogeneity (I^2^ = 90.0% and 91.8%, respectively). Moreover, the effect value, SMD, was significantly higher in the NASH group than in the NAFL group (*p* = 0.036). However, subgroup analysis showed no difference in the effect values of fetuin-A for plasma and serum samples (*p* = 0.282), and with respect to RACE (*p* = 0.155). Nonetheless, the effect values of fetuin-A in relation to storage methods were significantly different, and the effect values of fetuin-A according to frozen storage temperature were higher (*p* = 0.045) (Table 3). On the other hand, taking the studies which compared fetuin-B concentrations between patients with NAFLD and the controls, it was found that fetuin-B concentrations were higher in patients with NAFLD than in the controls, with low effect (SMD = 0.18, 95% CI: 0.02 to 0.33, *p* = 0.023), and moderate heterogeneity (I^2^ = 62.3%).

**Figure 3:**
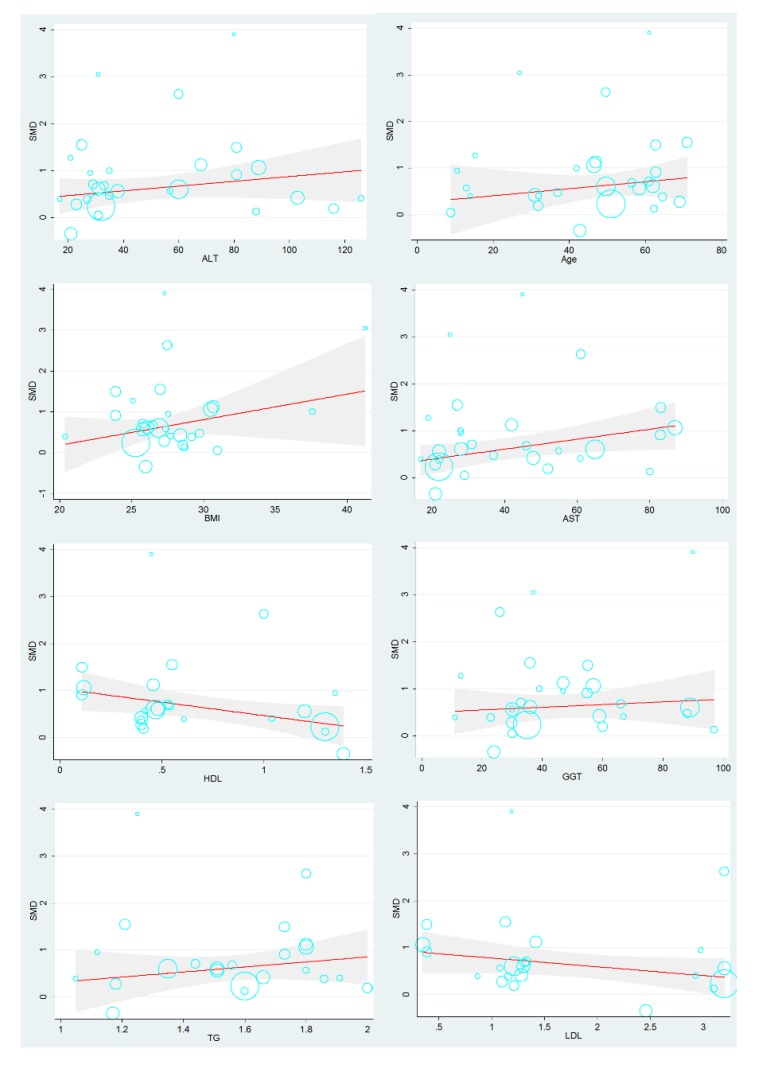
Dose-response relationships between clinical parameters of fetuin-A and SMD value outcomes based on data from each study. Each data point overlaps into a circle. The size of a circle represents the weight of the corresponding data point, and the larger the circle, the greater the impact. NAFLD, non-alcoholic fatty liver disease; AST, aspartate aminotransferase; ALT, alanine aminotransferase; GGT, glutamyl transpeptidase; LDL, low-density lipoprotein; HDL, high-density lipoprotein; TG, triglycerides; BMI, Body Mass Index; SMD, standardized mean difference.

**Figure 4:**
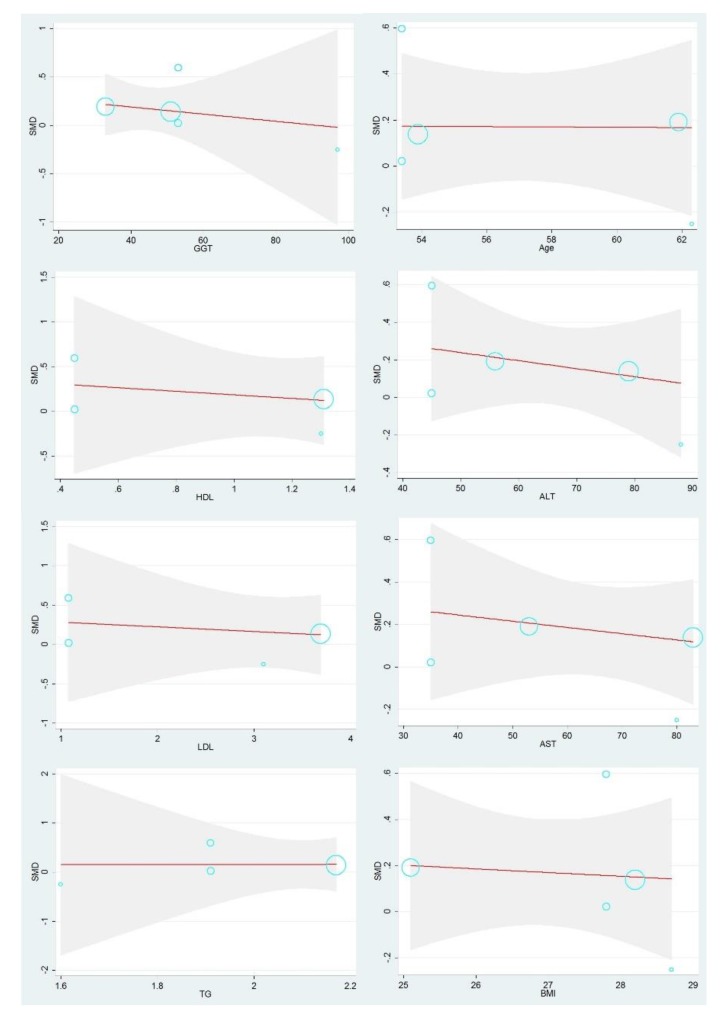
Dose-response relationships between clinical parameters of fetuin-B and SMD value outcomes based on data from each study. Each data point overlaps into a circle. The size of a circle represents the weight of the corresponding data point, and the larger the circle, the greater the impact. NAFLD, non-alcoholic fatty liver disease; AST, aspartate aminotransferase; ALT, alanine aminotransferase; GGT, glutamyl transpeptidase; LDL, low-density lipoprotein; HDL, high-density lipoprotein; TG, triglycerides; BMI, Body Mass Index; SMD, standardized mean difference.

Additionally, the results of meta-regression analysis (with respect to Age, AST, BMI, ALT, GGT, TG, LDL, and HDL) of fetuin-A and fetuin-B are shown in Figure 3 and Figure 4, respectively. As regards to fetuin-A and NAFLD, the following clinical characteristics had a positive effect on the SMD: BMI (β BMI = 0.0636, SE = 0.0437, *p* = 0.158), age (β age = 0.0057, SE = 0.0090, *p* = 0.529), AST (β AST = 0.0052, SE = 0.0077, *p* = 0.502), ALT (β ALT = 0.0018, SE = 0.0054, *p* = 0.734), GGT (β GGT = 0.0031, SE = 0.0074, *p* = 0.674), and TG (β TG = 0.0321, SE = 0.6628, *p* = 0.962). However, subsequent meta-regression analysis revealed a negative effect of LDL (β LDL = −0.0976, SE = 0.1854, *p* = 0.604) and HDL (β HDL = −0.4701, SE = 0.4258, *p* = 0.282) on SMD. Figure 3 shows each data point overlapping into a circle as illustrated by meta-regression models with 95% CIs. The size of a circle represents the weight of the associated data point such that the larger the circle the greater the impact.

Nonetheless, as regards fetuin-B and NAFLD, the following clinical characteristics had a negative effect on SMD: BMI (β BMI = −0.0374, SE = 0.1057, *p* = 0.747), age (β age = −0.0236, SE = 0.0316, *p* = 0.509), ALT (β ALT = −0.0080, SE = 0.0069, *p* = 0.331), AST (β AST = −0.0056, SE = 0.0058, *p* = 0.406), GGT (β GGT = −0.007, SE = 0.0007, *p* = 0.406), LDL (β LDL = −0.1100, SE = 0.1519, *p* = 0.544) and HDL (β HDL = −0.3586, SE = 0.4076, *p* = 0.472). However, subsequent meta-regression analysis revealed a positive effect of TG on SMD (β TG = 0.5838, SE = 0.9718, *p* = 0.609).

Sensitivity analysis showed that there was a small change in SMD and the corresponding 95% CI when each study was removed in turn, indicating that the current overall meta-analysis data were relatively stable. Publication bias was not reported for fetuin-B since the number of fetuin-B studies was less than 10. However, there was lack of symmetry in the shape of the Egger’s funnel plot for fetuin-A, and the Egger’s test was significant (t = 2.32, *p*-value = 0.028), implying that there may be publication bias for this comparison.

## 4. Discussion

This study has confirmed that there may be a relationship between fetuin-A, fetuin-B, and NAFLD. Specifically, there were higher concentrations of fetuin-A and fetuin-B in NAFLD patients than in the controls. The effect size of elevated fetuin-A concentrations on NAFLD was strong (SMD = 0.83), thus supporting the previous hypothesis that there are raised levels of fetuin-A in NAFLD. In addition, subgroup analysis showed that the effect value of fetuin-A concentrations was significantly higher on the NASH patients than on the NAFL patients, which may indicate that fetuin-A played an important role in the pathophysiological process of NAFL to NASH. This inference could not be made on fetuin-B due to a lack of relevant studies on the relationship between fetuin-B concentrations and NASH. Therefore, the changes of fetuin-B in NASH should be studied accordingly in the future. However, subgroup analysis showed no difference in the effect values of fetuin-A for plasma and serum samples, and with respect to RACE, whereas the effect values of fetuin-A in relation to storage methods were significantly different, and the effect values of fetuin-A according to frozen storage temperature were higher. Thus, these findings suggest that the effects of fetuin-A were stable in relation to sample sources, storage methods, and race. However, future research should try to use cold chain to transport and store fetuin-A. Noteworthy, similar results were found between fetuin-A concentrations and other diseases. For example, fetuin-A levels were increased in other glucose and lipid metabolism disorders such as type 2 diabetes mellitus and metabolic syndrome. Although NAFLD and these other diseases are similarly identified as chronic diseases, the role of fetuin-A in NAFLD is likely to be more complex.

**Figure 5:**
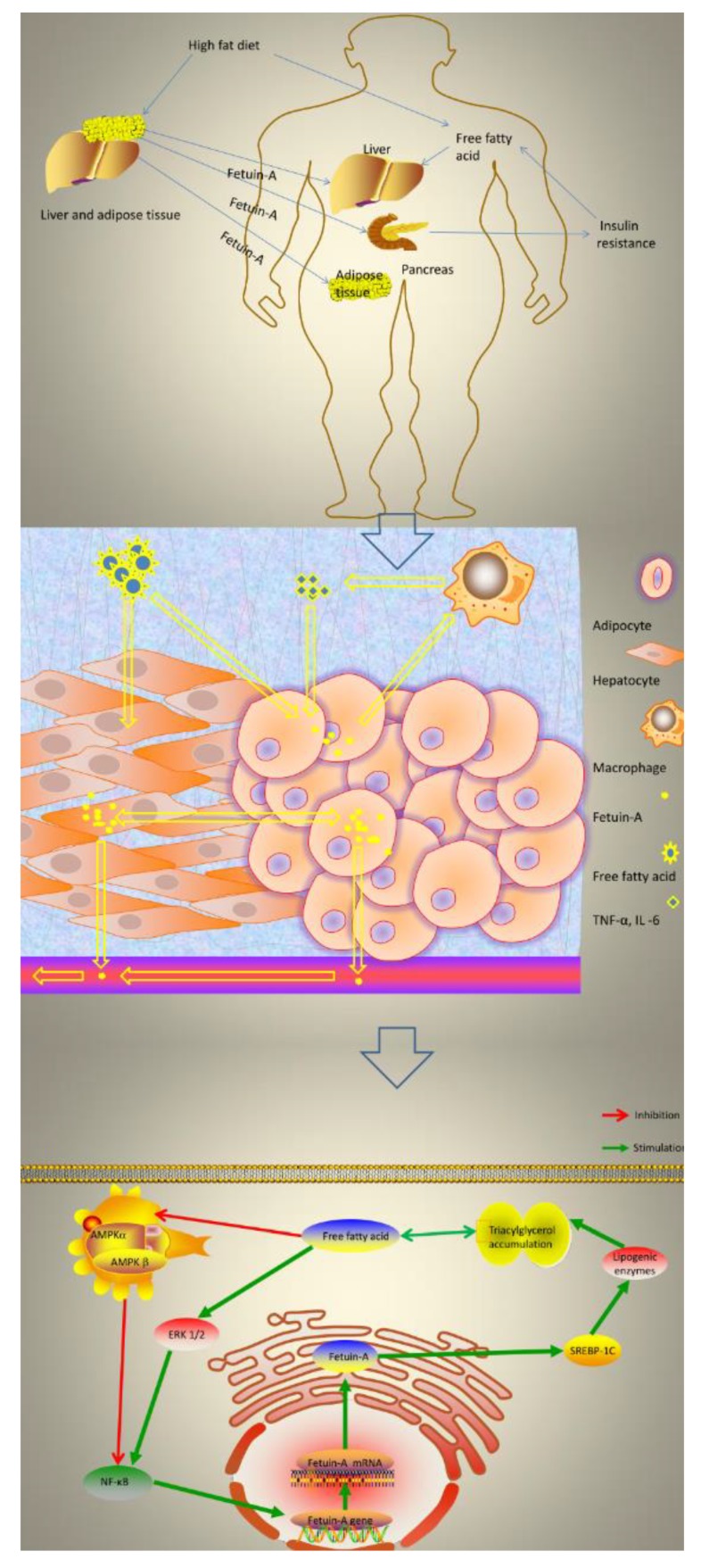
Summary of the hypothetical mechanism of fetuin-A in NAFLD. Figure 5 Schematic representation of the effects of fetuin-A on glucolipid metabolism signaling cascade in NAFLD that explain positive effects of these factors on glucolipid control. NAFLD, non-alcoholic fatty liver disease; IL -6: Interleukin-6; TNF-α: Tumor Necrosis Factor; AMPK: AMP-activated protein-kinase; NF-κB, nuclear factor κB; ERK 1/2, Extracellular signal-regulated kinases 1 and 2; SREBP- 1C, sterol regulatory element-binding protein-1c.

Notwithstanding, a recent study showed some hypothetical mechanisms about the relationship between fetuin-A and NAFLD. Specifically, that study suggested that a high-fat diet would bring excessive free fatty acids and cause hepatocytes and adipocytes to secrete fetuin-A, which can send chemical attraction signals and induce macrophages to infiltrate into adipose tissue [41]. In this regard, active macrophages increase the secretion of inflammatory cytokines, such as TNF-α and IL-6, which can further aggravate hepatocyte steatosis. Furthermore, existing data suggested that free fatty acids can increase the activity of nuclear factor κB (NF-κB) by inhibiting Adenosine 5-monophosphate-activated protein kinase (AMPK); and can also enhance the activity of NF-κB by activating ERK 1/2 [42]. Given that NF-κB plays an important role in up-regulating the expression of the fetuin-A gene and further increasing the level of fetuin-A mRNA, it brings about the overloaded fetuin-A in the cells, which may directly stimulate sterol regulatory element-binding protein-1c (SREBP-1C). It is generally acknowledged that the up-regulation of lipogenic enzymes by SREBP-1C is conducive to the accumulation of triacylglycerol [43]. An illustration of this series of hypotheses is shown in Figure 5 (by AK).

In addition, it has been confirmed from extensive studies of genetic epidemiology that polymorphisms in the gene encoding fetuin-A, the alpha-2-Heremans-Schmid glycoprotein (AHSG) gene, may affect glucose and lipid metabolism disorders’ pathophysiology progression in some higher-risk groups. Furthermore, metabolic syndrome and type 2 diabetes mellitus are concerned with the single nucleotide polymorphisms (SNPs) of two risk alleles in AHSG (rs2518136 and rs4917) [44]. Therefore, these results suggest that AHSG and SNPs are recognized as a susceptibility locus for glucose and lipid metabolism disorders, which can further induce NAFLD progression.

In addition, clinical and experimental studies concurred with the hypothesis of a relationship between NAFLD and fetuin-A. Besides, it is well known that fetuin-A is significantly increased in hepatocellular injury and mediates insulin resistance as well as impairs glucose tolerance [45]. Moreover, accumulating clinical evidence has shown that increased fetuin-A concentrations in centripetal obesity individuals may lead to the development of NAFLD in this group [45].

Although previous studies have shown that fetuin-A and fetuin-B may be affected by other clinical parameters in NAFLD, this study has shown that fetuin-A might be a potential predictor of the occurrence of NAFLD, independent of age, AST, BMI, ALT, GGT, TG, LDL, and HDL. However, HDL and LDL had no significant influence on the effect size of fetuin-A in NAFLD.

Considering that increased activity of GGT, AST, and ALT generally indicates chronic and progressive hepatitis lesions, it is no wonder that GGT, AST, and ALT showed a positive influence on NAFLD [46]. As regards TG, Verras and colleagues reported that serum fetuin-A levels were significantly positively correlated with serum TG, even after controlling for sex and age, and this is in agreement with the findings of this study [47]. As for BMI and age, a recent study of genetic epidemiology indicated that BMI was significantly related to fetuin-A gene AHSG rs2248690 in an Asian population. In addition, Marhaug and colleagues found that fetuin-A levels were significantly higher in adult populations than in children and adolescents [48].

Despite the reasons for the negative effects of the levels of LDL and HDL found in this study, not being well known, it is common for LDL and HDL cholesterols to play a counterproductive role and a protective role, respectively, in NAFLD and other metabolic diseases. Thus, it is likely that LDL levels might be affected by fetuin-A. This phenomenon is relevant to the impairment of SIRT1 and AMPK energy sensors, in which fetuin-A may play an intermediary role in lipid-induced hepatic fat lesions [49]. Further, this may be connected to the reduction of LDL secreted directly into the blood after hepatic fat lesions [50]. However, these assumptions are based on cross-sectional analyses and do not imply established causality. Therefore, it is of great importance to perform prospective studies to evaluate the effect of fetuin-A on these indicators in patients with NAFLD at different stages.

However, unlike for fetuin-A, there was a negative linear relationship between fetuin-B and age, AST, BMI, ALT, GGT, HDL, and LDL, but a positive linear relationship between fetuin-B and TG. Even though these results seem beyond our understanding, previous studies have shown that there was a great positive linear relationship between the natural progression of fetuin-B in NAFLD and hepatic steatosis. Therefore, this may explain why the relationship between these indicators and fetuin-B was different from that between these indicators and fetuin-A. Although there may be a negative correlation between fetuin-B and liver fibrosis, this study did not consider exploring sources of heterogeneity with respect to liver fibrosis because the liver fibrosis scores of the patients varied considerably across the eligible studies. Thus, the relationship between fetuin-B and liver fibrosis needs to be further studied [17]. In addition, due to the small number of studies included in the meta-regression analysis, the statistical capacity may be limited. Therefore, these results need to be understood with caution.

This meta-analysis has some limitations. First, these findings were obtained from case-control studies, which cannot make causal inferences. Therefore, prospective studies should be considered in the future to explore the relationship between fetuin-A, fetuin-B, and NAFLD at different stages. Second, confounding factors that may affect fetuin-A and fetuin-B concentrations, such as race and cigarette smoking, were not analyzed in this study because these were not reported in the eligible studies. Third, studies on fetuin-A and fetuin-B concentrations between NAFLD patients and the controls showed high heterogeneity, which is not surprising given the large differences with respect to the study area and study population. Therefore, future original studies should consider making these investigations on homogeneous samples.

## 5. Conclusions

To sum up, the findings of this study showed that there were significantly higher fetuin-A and fetuin-B concentrations in NAFLD patients than in the controls. Thus, fetuin-A and fetuin-B might be potential predictors of the occurrence of NAFLD. Notably, fetuin-A may play an important role in the pathophysiological process of NAFL to NASH. These findings, therefore, provide a rationale for evaluating fetuin-A and fetuin-B in the pathophysiological process of NAFLD, and might open up new perspectives in early diagnosis, identification of novel biomarkers, and providing novel targets for pharmacological interventions.

## Figures and Tables

**Table 1:** Characteristics of the studies included for the meta-analysis of fetuin-A, fetuin-B, and NAFLD.

Study		Material	Country	NOS	Male Gender, *n* (%)	BMI	Mean Age	Study Type	Diagnosis of NAFLD	Overnight Fasting Blood	Methods	Frozen
**(Ballestri et al., 2013)**	[14]	Serum	Italy	7	20(69.0)	29.2 ± 5.0	64.5 ± 10.5	Case-control	Ultrasonography	Yes	ELISA	NR
**(Celebi et al., 2015)**	[7]	Plasma	Turkey	8	69(100.0)	28.57 ± 3.40	31.8 ± 5.6	Case-control	Liver biopsy specimens scored	Yes	ELISA	−80 °C
**(Cui et al., 2017)**	[15]	Serum	China	6	58(73.4)	26.0 ± 3.0	42.8 ± 10.8	Case-control	Ultrasonography	Yes	ELISA	NR
**(Dogru et al., 2013)**	[16]	Plasma	Turkey	7	31(100.0)	28.4 ± 2.97	31.0 ± 3.9	Case-control	Liver biopsy specimens scored	Yes	ELISA	−80 °C
**(Ebert et al., 2017)**	[17]	Serum	Germany	6	52(51.4)	28.7 ± 6.8	62.3 ± 16.4	Case-control	Ultrasonography	Yes	ELISA	NR
**(Şiraz Ü et al., 2017)**	[18]	Serum	Turkey	7	4(50.0)	20.4 ± 5.6	14.0 ± 3.7	Case-control	Ultrasonography	Yes	ELISA	−80 °C
**(Haukeland et al., 2010)**	[19]	Plasma	Norway	8	77(62.0)	23.9 ± 3.0	62.7 ± 7.5	Case-control	Liver biopsy specimens scored	No	ELISA	−80 °C
**(Haukeland et al., 2012)**	[20]	Plasma	Norway	8	67(60.0)	30.5 ± 4.3	46.5 ± 11.6	Case-control	Liver biopsy specimens scored	No	ELISA	−80 °C
**(El-Ashmawy and Ahmed, 2019)**	[21]	Serum	Egypt	8	55(52.4)	27.8 ± 4.1	53.4 ± 9.2	Case-control	Ultrasonography	Yes	ELISA	NR
**(Kahraman et al., 2013)**	[5]	Serum	Turkey	6	55(23.1)	37.6 ± 1.2	41.9 ± 0.9	Case-control	Liver biopsy specimens scored	No	ELISA	4 °C
**(Kurtoglu et al., 2015)**	[22]	Serum	Turkey	5	4(50.0)	25.1 ± 3.0	15.3 ± 3.7	Case-control	Ultrasonography	No	ELISA	NR
**(Lebensztejn et al., 2014)**	[23]	Serum	Poland	7	12(63.3)	27.4 ± 7.3	13.0 ± 3.9	Case-control	Ultrasonography	No	ELISA	NR
**(von Loeffelholz et al., 2016)**	[24]	Plasma	Germany	8	35(46)	27.3 ± 1.1	61.0 ± 2.0	Case-control	Liver biopsy specimens scored	Yes	ELISA	−80 °C
**(Li et al., 2018)**	[25]	Serum	China	7	283(36.2)	28.2 ± 3.1	53.9 ± 7.0	Case-control	Ultrasonography	Yes	ELISA	NR
**(Lin et al., 2018)**	[26]	Serum	China	5	398(65.3)	25.1 ± 3.0	61.9 ± 7.3	Case-control	Ultrasonography	No	ELISA	NR
**(Mondal et al., 2018)**	[27]	Serum	India	8	26(56.5)	27.5 ± 6.2	49.6 ± 12.2	Case-control	Ultrasonography	Yes	ELISA	−80 °C
**(Nascimbeni et al., 2018)**	[2]	Serum	Italy	7	34(76)	27.3 ± 19.1	69.0 ± 11.7	Case-control	Ultrasonography	Yes	ELISA	NR
**(Nascimbeni et al., 2015)**	[28]	Serum	Italy	7	62(78.2)	27.0 ± 4.0	71.0 ± 10.0	Case-control	Ultrasonography	Yes	ELISA	NR
**(Ou et al., 2012a)**	[29]	Serum	China	7	51(56.7)	26.1 ± 3.1	62.0 ± 10.0	Case-control	Ultrasonography	Yes	ELISA	NR
**(Ou et al., 2012b)**	[30]	Serum	China	7	25(55.6)	25.8 ± 3.0	61.0 ± 10.0	Case-control	Ultrasonography	Yes	ELISA	NR
**(Pampanini et al., 2018)**	[31]	Serum	Sweden	6	5(55.6)	31.0 ± 9.0	8.9 ± 2.3	Case-control	Ultrasonography	Yes	ELISA	NR
**(Rametta et al., 2014)**	[32]	Plasma	Italy	8	106(77.3)	26.9 ± 3.4	49.7 ± 12.1	Case-control	Liver biopsy specimens scored	Yes	ELISA	−80 °C
**(Reinehr and Roth, 2008)**	[33]	Serum	USA	8	9(75.0)	27.6 ± 3.8	10.6 ± 2.8	Case-control	Ultrasonography	Yes	ELISA	−80 °C
**(Roshdy et al., 2015)**	[34]	Serum	Egypt	5	24(77.4)	41.26 ± 11.2	27.0 ± 2.7	Case-control	Ultrasonography	No	ELISA	NR
**(Sato et al., 2015)**	[35]	Serum	Japan	6	151(86.3)	26.5 ± 3.6	56.4 ± 6.9	Case-control	Ultrasonography	Yes	ELISA	−80 °C
**(Sonmez et al., 2015)**	[36]	Serum	Turkey	8	17(70.0)	27.72 ± 1.99	32.0 ± 6.0	Case-control	Liver biopsy specimens scored	Yes	ELISA	−80 °C
**(Tanoglu et al., 2015)**	[37]	Serum	Turkey	5	35(83.3)	29.72 ± 3.91	37.0 ± 6.0	Case-control	Liver biopsy specimens scored	No	ELISA	NR
**(Wong et al., 2015)**	[38]	Serum	China	7	143(54.4)	25.3 ± 3.4	51.0 ± 9.0	Case-control	Ultrasonography	Yes	ELISA	NR
**(Yilmaz et al., 2010)**	[39]	Serum	Turkey	8	46(46.5)	30.7 ± 4.9	47.0 + 9.0	Case-control	Ultrasonography	Yes	ELISA	−80 °C
**(Zhu et al., 2017)**	[40]	Serum	China	7	54(58.7)	25.8 ± 2.5	58.4 ± 10.9	Case-control	Ultrasonography	No	ELISA	NR

NOS, Newcastle-Ottawa Scale; BMI, Body Mass Index; ELISA, enzyme-linked immunosorbent assay; NR, not report; NAFLD, non-alcoholic fatty liver disease.

**Table 2:** Characteristics of clinical parameters of the studies included for the meta-analysis of fetuin-A, fetuin-B, and NAFLD.

Study		AST (IU/mL)	ALT (IU/mL)	GGT (IU/mL)	TG (mmol/L)	LDL (mmol/L)	HDL (mmol/L)	HOMA-IR
**(Ballestri et al., 2013)**	[14]	22.00	27.00	23.00	1.86	1.16	0.40	1.50
**(Celebi et al., 2015)**	[7]	52.00	116.00	60.00	2.00	1.21	0.41	3.50
**(Cui et al., 2017)**	[15]	21.00	21.00	24.00	1.17	2.46	1.39	3.27
**(Dogru et al., 2013)**	[16]	48.00	103.00	59.00	1.66	1.28	0.40	3.35
**(Ebert et al., 2017)**	[17]	80.00	88.00	97.00	1.60	3.10	1.30	NR
**(Şiraz Ü et al., 2017)**	[18]	17.00	17.00	11.00	1.05	0.87	0.61	NR
**(Haukeland et al., 2010)**	[19]	83.00	81.00	55.00	1.73	0.39	0.11	2.11
**(Haukeland et al., 2012)**	[20]	87.00	89.00	57.00	1.80	0.35	0.12	2.21
**(El-Ashmawy and Ahmed, 2019)**	[21]	35.00	45.00	53.00	1.91	1.08	0.45	4.90
**(Kahraman et al., 2013)**	[5]	28.00	35.00	39.00	NR	NR	NR	NR
**(Kurtoglu et al., 2015)**	[22]	19.00	21.00	13.00	NR	NR	NR	NR
**(Lebensztejn et al., 2014)**	[23]	55.00	57.00	30.00	1.80	1.08	0.42	3.67
**(von Loeffelholz et al., 2016)**	[24]	45.00	80.00	90.00	1.25	1.19	0.45	3.30
**(Li et al., 2018)**	[25]	83.00	79.00	51.00	2.17	3.69	1.31	4.09
**(Lin et al., 2018)**	[26]	53.00	56.00	33.00	NR	NR	NR	3.23
**(Mondal et al., 2018)**	[27]	61.00	60.00	26.00	1.80	3.20	1.00	1.10
**(Nascimbeni et al., 2018)**	[2]	21.00	23.00	30.00	1.18	1.10	0.40	1.80
**(Nascimbeni et al., 2015)**	[28]	27.00	25.00	36.00	1.21	1.13	0.55	1.91
**(Ou et al., 2012a)**	[29]	28.00	31.00	36.00	1.51	1.30	0.48	1.13
**(Ou et al., 2012b)**	[30]	31.00	29.00	33.00	1.44	1.33	0.53	2.90
**(Pampanini et al., 2018)**	[31]	29.00	31.00	30.00	NR	NR	NR	NR
**(Rametta et al., 2014)**	[32]	65.00	60.00	89.00	1.35	1.21	0.47	2.50
**(Reinehr and Roth, 2008)**	[33]	28.00	28.00	47.00	1.12	2.98	1.35	3.90
**(Roshdy et al., 2015)**	[34]	25.00	31.00	37.00	NR	NR	NR	NR
**(Sato et al., 2015)**	[35]	46.00	33.00	66.00	1.56	1.31	0.54	NR
**(Sonmez et al., 2015)**	[36]	61.00	126.00	67.00	1.91	2.93	1.04	3.92
**(Tanoglu et al., 2015)**	[37]	37.00	35.00	88.00	NR	NR	NR	NR
**(Wong et al., 2015)**	[38]	22.00	32.00	35.00	1.60	3.20	1.30	2.50
**(Yilmaz et al., 2010)**	[39]	42.00	68.00	47.00	1.80	1.42	0.46	3.80
**(Zhu et al., 2017)**	[40]	22.00	38.00	30.00	1.51	3.20	1.20	NR

AST, aspartate aminotransferase; ALT, alanine aminotransferase; GGT, glutamyl transpeptidase; LDL, low-density lipoprotein; HDL, high-density lipoprotein; TG, triglycerides; and HOMA-IR, insulin resistance index.

**Table 3:** Subgroup analyses of fetuin-A between NAFLD participants and controls.

	Number of Studies	SMD (95% CI)	Z	*p*-Value	Heterogeneity		
					Q Statistic (DF; *p*-Value)	τ^2^	I^2^
**Material**							
**Plasma**	7	1.0426 [0.6067; 1.4786]	4.69	0.000	68.21 6 < 0.0001	0.30	91.20%
**Serum**	21	0.7546 [0.4626; 1.0466]	5.07	0.000	224.35 20 < 0.0001	0.40	91.10%
**Study region**							
**Asia race**	15	0.6741 [0.3594; 0.9888]	4.20	0.000	160.42 14 < 0.0001	0.33	91.30%
**Caucasian race**	13	1.0238 [0.6580; 1.3897]	5.49	0.000	119.20 12 < 0.0001	0.38	89.90%
**Frozen**							
**Yes**	14	0.9508 [0.5704; 1.3312]	4.9	0.000	163.53 13 < 0.0001	0.46	92.10%
**No**	14	0.5940 [0.3070; 0.8809]	4.06	0.000	108.10 13 < 0.0001	0.24	88.00%

Subgroup analyses are performed to compare the concentration of each fetuin-A between NAFLD and the controls. Heterogeneity was quantified using I^2^ and its significance was tested using the Q statistics. SMD, standardized mean difference; DF, degrees of Freedom; NAFLD, non-alcoholic fatty liver disease.

## Supplementary Materials

The following are available online at https://www.mdpi.com/1660-4601/17/8/2735/s1, **Supplementary File**: Search strategies. Details of the search strategy.
